# Du Varicocele, Et En Particulier De La Cure Radicale De Cette Affection

**Published:** 1841-05

**Authors:** 


### REVIEWS.

---

Art. I.—*Du Varicocele, et en particulier de la Cure Radicale de Cette Affection ; par* H. Landouzy, *interne a l'Hotel-Dieu, membre de la Societe Anatomique et de la Societe Medicale D'Observation, &c.*

Essay on Varicocele, particularly on the Radical Cure of this Disease. By H. Landouzy. 8vo. pp. 120, with a lithographic plate. Paris: 1838.

A monograph on varicocele is an acknowledged desideratum in our surgical literature. No sufficient work has ever been issued from the British or American press on the subject, and if it be true, as M. Landouzy seems to think it is, that six-tenths of the male sex are afflicted with this singular malady, a careful analysis of the contents of his essay will not, it is believed, be unprofitable.

The term varicocele, compounded out of the Greek and Latin, is employed to desiginate a dilated condition of the veins of the scrotum and spermatic cord, and is regarded by the author of the present essay as synonymous with the word circocele and varicose hernia. By some of the older writers, such as Celsus, Murray, Waitz, and Most, the affection is described under the appellation of circocele, while Callisen, Boyer, Delpech, Sir Astley Cooper, Breschet, Blandin, Velpeau, and other modern surgeons, use the name varicocele to denote the varicose enlargements of the scrotum, no matter what part of it they may implicate.

Although this disease occurs exclusively in the male, yet there is occasionally in the pudendal lips of the female a varicose disposition which corresponds in its essential features to the varicose enlargement of the scrotum; the veins of the ovaries and of the round ligament of the uterus are also liable to a species of dilatation which may be said to resemble that of the spermatic cord. Still the affection is very different in the two sexes; for while in the female it is caused either by pregnancy or some lesion of the abdomen, and is therefore usually of a transient nature, disappearing with the removal of the cause by which it was induced, in the male it is not only more permanent, but is apt to be attended, if allowed to proceed unrestrained, by very serious consequences, especially to the corresponding testicle.

Varicocele may appear at any period of life, in the young as well as in the old. Professor Delpech, of Montpelier, thought it was almost exclusively confined to adults or aged people; an opinion which is directly opposed to that of M. Landouzy, who, in twenty-seven cases in which this subject was noticed, ascertained that seven occured between the ages of nine and fifteen; seventeen between fifteen and twenty-five; and three between twenty-five and thirty-five. Thus, if this statement can be relied upon, and we can see no reason to doubt it, it must be concluded that the malady in question is most common, by far, in young subjects within the first ten years from the time of puberty, or during the period of the greatest excitement of the genital system.

The development of this disease appears to be greatly influenced by the anatomical disposition of the parts, as the dependent situation of the spermatic veins, their length, the feebleness of their parietes, relatively to the course which they are obliged to traverse, the absence of valves, and especially the frequent alternations of repletion and vacuity to which they are exposed in the various attitudes and movements of the body. Another cause which likewise powerfully contributes to the production of varices, is the enormous quantity of veins which, under the name of the pampiniform plexus, give rise to the veins of the testicle. In the normal state, andparticularly when the scrotum is contracted, this venous packet does not appear to be very large; but as soon as they are in the slightest degree enlarged, or even when the parts are merely relaxed by heat, a good idea of it is afforded by the immense number of venous branches which surmount and encircle the testicle and epididymis. Besides these circumstances, our author supposes, and very justly as we think, that the pressure of the column of blood in these vessels must, according to a well known law of hydrostatics, exert no little influence in the formation of varicocele. J. L. Petit was inclined to ascribe considerable importance to the weight of the testicle, which has a tendency, he imagines, to obliterate the vessels at the external ring, and thus render the return of their contents more difficult.

Pathologists have long been familiar with the fact that varicocele is much more frequent on the left side than on the right, and many ingenious notions have been offered in explanation of it. Morgagni originally suggested the idea, which has since been adopted by many others, that it was owing to the more dependent situation of the left testicle, and the greater length of the left spermatic vein, which, instead of pouring its contents into the inferior cava, like the right, terminates in the left renal, nearly at a right angle with the current of the blood from the kidney. Others have attributed the greater frequency to the compression made upon the spermatic vessels of the left side, by the fœcal matters accumulated in the iliac portion of the colon. Such, among others, is the opinion of Callisen and of J. L. Petit. According to M. Landouzy, however, this influence is greatly overrated, as out of seventeen patients whom he carefully examined in reference to this subject only a single one was found to be habitually affected with constipation of the bowels. He also thinks that the opinion of Dr. Lenoir, of Paris, who attempts to explain the circumstance in question by the narrowing of the left inguinal ring, from the frequent contraction of the abdominal muscles, although plausible, is without foundation; a view in which we entirely concur with him. Be the cause, however, what it may, the fact is undeniable that the disease is generally found on the left side, as we have pointed out in another publication. In upwards of one hundred cases examined by Breschet, of Paris, only one occurred on the right side. With this result the experience of nearly every practitioner must coincide.

The exciting causes of this affection the author arranges under two heads, under those, namely, which facilitate the afflux of blood to the genital organs, and those which prevent its return to the heart. These two orders of causes may exist separately, or occur conjointly in the same individual. In the first may be enumerated all venereal excesses, masturbation, violent passions, riding, dancing, severe exercise on foot; in short, whatever has a tendency to invite the blood constantly to the lower parts of the body. Under this head, may also be included violent contusions of the scrotum and inflammation of the testicle, whether essential or symptomatic, which, by keeping up irritation and a considerable afflux of blood, may at length produce dilatation of the vessels, or render them more sensible to the causes of accidental enlargement.

The causes of the second order are much more frequent, since they embrace every thing that opposes an obstacle to the return of the blood to the heart. Thus, tumors developed in the abdomen, whatever may be their seat or nature, may compress, either directly or indirectly, the spermatic vessels, and thus occasion varicocele. In the inaugural dissertation of M. Brixoux, published in 1837, a very interesting case is recorded in which the disease seems to have been produced by this cause. The patient, who had been operated on at la Charitie according to Velpeau's method, died in consequence of some other disease, which, upon dissection, was found to be a very large encephaloid tumor, situated in the left ileo-lumbar region, and compressing the vein of the corresponding testicle.

Inguinal and crural herniæ, whether congenital or accidental, may also oppose the venous circulation of the scrotum and of the cord, either by their direct compression upon the vessels, or by the manner in which they diminish the internal abdominal ring. The same effect may result from swelling of the lumbar ganglions, from scirrhous tumors along the spermatic cord, from hydrocele, and other kindred affections.

Hypochondriasis and melancholy have been enumerated as causes of varicocele; but without, as our author thinks, sufficient reason. The same is true of what Sir Astley Cooper says about the fatty accumulations of the omentum and mesentery, which, by compressing the spermatic vessels, interrupt, he supposes, the return of blood to the abdomen. According to Landouzy, considerable mischief occasionally results from the wearing of ill-constructed trusses, improperly applied suspensory bags, tight waistbands, or even tight pantaloons; at all events, there is reason to believe that if the disease is not thus induced, it is frequently kept up, increased, or aggravated.

We have already alluded to masturbation and venereal excesses as so many causes of the disease under consideration; and there can be no doubt that they are much more frequently concerned in its production than is generally imagined. Indeed, we are fully persuaded, from considerable experience, that this is the case. How these causes act in developing this affection admits of ready explanation. Their tendency is not only to determine an abundant afflux of blood to, and consequent congestion in, the genital organs, but to produce more or less fatigue in the muscles of those parts, especially in the cremaster and dartos, together with a loss of nervous innervation, which diminish their power and contractile energy. The testicle being thus insufficiently sustained sinks down, by its own weight, into the scrotum, which, with the spermatic vessels, is thereby kept in a state of constant relaxation. Heat acts in a similar manner, and produces similar results. Hence varicocele is much more frequent in hot than in temperate climates.

Finally, varicocele appears to be occasionally hereditary. Professor Blandin, of Paris, in an able article on this disease, in the "Dict. de Medicine et Chirurgie Pratiques," refers to three brothers with whom he was personally acquainted, who were all exempted from military duty on account of the existence of this malady: the father was similarly affected. An analogous case is mentioned in an inaugural dissertation published at Paris in 1837.

The next topic commented upon is the co-existence of varicocele with other affections, especially varices of the inferior extremities. In fifteen cases, in which careful inquiry was made in reference to this point, the occurrence was noticed only once. On the other hand, in twenty-one persons affected with varicose enlargement of the legs, the spermatic veins were found to be perfectly free from disease. These data, although insufficient, would lead us to conclude that there is rarely any connection, and then not necessarily so, between the two maladies in question. The same is true of hemorrhoids.

We now come to speak of the symptoms and progress of varicocele. In the early stage, the affection is generally announced by a sense of weight in the testicle, extending to the groin or even the lumbar region; unusual pain and dragging in the course of the spermatic cord, and elongation of the scrotum, which is soft, pendulous, and apt to increase very much in bulk from heat or long walks. Another circumstance which is remarked at this period, is the frequent handling of the parts by the patient, to place them in a more favorable position, and to support them the better by means of his clothes. As the disease progresses, exercise becomes painful, the patient is easily fatigued, the features are visibly altered, the surface is bathed with perspiration on the slightest exertion, and the affected organ has to be constantly supported by a suspensory bag. The pain, which is commonly of a dull, aching character, often extends to the back and kidney; in some instances colic is experienced; in others, there is a sense of weight in the anus; and in one patient, observed by M. Landouzy, the penis was affected with smarting sensations, attended with scalding in discharging his urine. Another symptom, which the author says is never wanting, and which writers have hitherto neglected to mention, is a remarkable increase of the cutaneous secretion of the scrotum of the affected side.

When fully developed, the disease presents itself under the form of a soft, fluctuating, knotty tumor, extending from the upper border of the testicle to the inguinal ring. The enlargement has neither the regular outline and elastic feel of hydrocele, nor the firmness and globular aspect of sarcocele, nor the compact consistence of inguinal hernia. In case it is very voluminous, it usually occupies the whole of the left side of the scrotum, from the base of the external ring. The subcutaneous veins, under these circumstances, are unusually distinct, forming a very prominent superficial net-work. Indeed, it not unfrequently happens that the vessels which are ramified over the surface of the scrotum equal in volume the femoral vein of the adult, as in one of the cases mentioned by our author.

As to the spermatic veins themselves, they frequently acquire nearly as large a volume, forming a mass which may be justly compared to a bundle of irregularly rolled up cords, to a cluster of earth-worms, or to the intestines of a chicken. On percussing the tumor attentively, we perceive very distinctly that it contains more vessels than in the normal state, and that it is difficult to distinguish the vas deferens in the midst of the inextricable net-work. Some of the vessels present the feel of membranous tubes, with soft fluctuating parietes, while others are quite hard, owing to their being partly obliterated or filled with coagulated fibrin.

Although the symptoms of this disease are usually well marked, yet it is sometimes liable to be confounded with other lesions. The affection for which it is most apt to be mistaken is inguinal hernia, especially that variety of it in which the omentum is concerned. The best way in such cases is to place the patient on his back, and hold up the scrotum until the vessels are entirely emptied of their contents; the finger is then applied against the external ring, and the patient requested to rise, when, if the disease be varicocele, the spermatic veins will immediately refill, whilst, if it be hernia, the bowel will be unable to descend.

The progress of this disease is usually slow, years elapsing before it occasions much suffering or inconvenience. In some instances, however, it increases with great rapidity, and gives rise to severe local disturbance, with more or less constitutional derangement. One of the most serious and unpleasant effects from this disease is atrophy of the testicle and epididymis, produced evidently by the pressure of the enlarged veins by which these organs are surrounded, as well as by those which enter into their composition, and which, in the more severe forms of the malady, are usually much increased in size. Celsus, it would appear, was already fully aware of this fact, which has been particularly noticed since by Pott, Callisen, and other surgeons. In thirteen of the cases recorded by our author, the left testicle was found to be more or less atrophied in nine. Occasionally, though rarely, this lesion exists simultaneously on both sides. In either case, it is generally attended with a gloomy and melancholy state of the mind, sometimes bordering upon alienation. Of this, the writer of this article has witnessed several instances.

It is seldom that opportunities are afforded for dissecting the parts concerned in varicocele. It is to be regretted, therefore, that the older surgeons, who were in the habit of extirpating the spermatic veins, or even of performing the operation of castration, for the relief of this disease, have left us no positive information respecting the pathological anatomy of it. Sir Astley Cooper, indeed, is almost the only writer who has said any thing on the subject. "On dissection," he observes, "all the veins are found to be dilated, and to have undergone such a degree of elongation, that the one situated along the course of the vas deferens descends much lower than even the testicle itself, which is thus placed, as it were, in front of it. It follows, moreover, from this elongation, that a considerable portion of the varicocele passes below the level of the organ in question. At the same time that these vessels are distended and rendered varicose, their coats are thickened to enable them to sustain the increased column of blood; or rather, this thickening is produced by the greater quantity of blood which flows in their *vasa vasorum*." In several cases in which we have carefully examined the condition of these vessels after death, we found them to be greatly convoluted, knotty, harder in some places than in others, and irregularly dilated, many of them being more than ten times the ordinary volume. Their parietes were very thick, dense, and rigid, almost of the nature of fibro-cartilage, at some points, and very much attenuated, soft, or rather brittle, at others. In cases of long standing, some of the veins are completely obliterated by adhesive inflammation, or by the formation of fibrinous concretions, imparting to the finger the sensation of so many solid bodies, pieces of cat-gut, or whipcord.

Having described the seat of varicocele, together with its causes, its symptoms, and its nature, the author at length arrives at the treatment, which he divides into palliative and radical. Regarded until recently as incurable, or as beyond the reach of any surgical operation, temporary relief was all that was ever looked for by the practitioner. This consisted chiefly in the wearing of an elastic suspensory bag, in the use of cold lotions, either simple or slightly astringent, to the scrotum night and morning, and in the strict avoidance of every thing calculated to favor a determination of blood to the spermatic vessels. To obtain full advantage from this treatment, the patient should refrain from dancing, horseback exercise, fatiguing walks, warm bathing, venereal indulgences, and protracted standing; which, as we have already seen, are so many causes of the malady under consideration.

In slight cases, this plan of treatment will doubtless answer very well; but when the disease is fully developed, progresses with unusual rapidity, is attended with much suffering, or threatens the destruction of the testicle, a radical cure is demanded. For this purpose various operations have been devised. With some of the ancients the actual cautery was a favorite remedy, especially when the affected veins were seated superficially; if, on the other hand, they were far removed from the surface, they first tied and afterwards extirpated them; an operation still resorted to by some of our modern surgeons. Gooch and other writers have reported cases of varicocele in which the suffering was so constant and intolerable, that nothing but castration could afford the patient any relief. In this country the spermatic artery has been repeatedly tied for the cure of this disease. The operation, we believe, was first performed in 1821 by Dr. Jameson, of Baltimore, who published an account of it a few years afterwards in the American Medical Recorder. In none of the cases in which he executed it did the patient suffer any inconvenience, either in regard to the integrity of the testis, or as respects the exercise of its functions. It is proper to state, however, that the operation is not only difficult of performance, but that it occasionally fails. Varicocele has also been cured by tying the spermatic veins. Successful cases of this operative procedure have been reported by Professor J. C. Warren, of Boston, and Dr. Stephen Brown, of New York.

Excision of the spermatic veins is another expedient which is sometimes resorted to for the cure of obstinate varicocele. Professor Warren, who seems to prefer this operation to all others, states that he has repeatedly executed it, that it always affords great relief, and that he has never found it necessary to repeat it. Whether others have been equally successful, we have no means of determining. To perform this operation the patient should be placed on his back, and the external parts carefully shaved. An assistant holding the vas deferens between his thumb and forefinger, the surgeon makes an incision from an inch and a half to two inches in length over the most prominent part of the tumor down to the veins which, bursting through the opening, are now to be seized with the forceps, and divided with the knife or scissors, at the upper and lower angles of the wound. The bleeding frequently ceases spontaneously; but should it be necessary ligatures are to be applied to as many orifices as may require them, when the edges of the wound should be approximated by one or more stitches and closed with adhesive plaster. In one instance, in which this operation was performed by Dr. Warren, a large quantity of blood was extravasated into the cellular substance of the scrotum, followed by inflammation, extensive sloughing, and loss of the testicle.

The best method, however, of effecting the radical cure of varicocele, according to our author, is that proposed by M. Breschet, of Paris. It is founded upon the anatomical arrangement of the part, that is, upon the facility with which the varicose vessels can be isolated from the spermatic artery and vas deferens, and afterwards compressed, so as to obliterate their caliber. This is effected by means of a forceps with flattened plates, which are worked by a screw. The pressure is applied in a gradual manner, but with sufficient force to destroy the life of the scrotum and of the affected vesself. The instrument is usually removed in from six to eight days; in the meanwhile the patient is kept on his back, cold lotions are applied to the scrotum, and the case treated upon general principles. When the slough is detached, the edges of the wound are approximated by adhesive straps, and the person is permitted to walk about. "The radical cure of varicocele," observes the author, "is thus obtained with the greatest certainty and without the slightest danger to the patient." In this way, M. Breschet is said to have operated successfully in more than one hundred cases, a number of which were witnessed by M. Landouzy, and are minutely detailed in the present essay. The average period required for a complete cure is twenty-three days.

Whether the method of Breschet, so much vaunted by our author, is really entitled to the praise which has been bestowed upon it, further experience alone can determine. In this country, it has not been sufficiently tried to enable us to form any opinion in regard to its value. The operation, it strikes us, must be exceedingly painful, so much so, as to prevent the majority of persons affected with varicocele from submitting to it, excepting as an ulterior resort. In speaking on this subject, Dr. Warren observes: "I have not been able to prevail with patients to keep on the screw long enough to effect the object wished for. The last to whom it was applied took off the screw a short time after its application, walked out of the hospital, and has not been heard of since."* A similar remark has been made by others.

It will not be necessary, after what has been said respecting the different operations that have been devised for the relief or radical cure of this disease, to describe the different methods of Velpeau, Davas, Fricke, Grossheim, and Rey-

*Surgical Observations on Tumors, p. 439: Boston, 1837.

naud, more especially as they must all be regarded, at least to some extent, as so many modifications of the plan proposed by M. Breschet. We shall, therefore, conclude the present notice by inserting an account of the operation of the late Sir Astley Cooper, which, on the whole, we prefer to any that has yet been suggested, especially when, as often happens, there is great relaxation of the scrotum, on a partial removal of which it is founded. The mode of performing it is as follows: "The patient being placed in a recumbent posture, the relaxed scrotum is drawn between the fingers; the testis is to be raised to the external ring by an assistant; and then the portion of the scrotum is removed by the knife or knife-scissors; but I prefer the former. Any artery of the scrotum which bleeds is to be tied; and a suture is then made to bring the edges of the diminished scrotum together. The patient should be kept for a few hours in the recumbent posture, to prevent any tendency to bleeding; and then a suspensory bag is to be applied, to press the testis upwards, and to glue the scrotum to the surface."*

"It is of no use," adds Sir Astley, "to remove a small portion of the scrotum, for from doing this I have failed. When the wound has healed, the varicocele is lessened, but not always entirely removed; but the pain and distressing sensations cease, if sufficient of the scrotum be removed." The operation is not painful, nor is it followed by any constitutional irritation. The intention of it is, as the reader will at once perceive, to afford a permanent support to the enlarged vessels, rendering it thereby unnecessary for the patient to wear a suspensory bandage.

S. D. G.

---

*Dunglison's American Medical Library, p. 301: Philadelphia, 1839.

